# Marine Sponge-Derived *Streptomyces* sp. SBT343 Extract Inhibits Staphylococcal Biofilm Formation

**DOI:** 10.3389/fmicb.2017.00236

**Published:** 2017-02-16

**Authors:** Srikkanth Balasubramanian, Eman M. Othman, Daniel Kampik, Helga Stopper, Ute Hentschel, Wilma Ziebuhr, Tobias A. Oelschlaeger, Usama R. Abdelmohsen

**Affiliations:** ^1^Institute for Molecular Infection Biology, University of WürzburgWürzburg, Germany; ^2^Institute of Pharmacology and Toxicology, University of WürzburgWürzburg, Germany; ^3^Department of Analytical Chemistry, Faculty of Pharmacy, Minia UniversityMinia, Egypt; ^4^Department of Ophthalmology, University Hospital WürzburgWürzburg, Germany; ^5^GEOMAR Helmholtz Centre for Ocean Research, RD3 Marine Microbiology, and Christian-Albrechts University of KielKiel, Germany; ^6^Department of Botany II, Julius-von-Sachs Institute for Biological Sciences, University of WürzburgWürzburg, Germany; ^7^Department of Pharmacognosy, Faculty of Pharmacy, Minia UniversityMinia, Egypt

**Keywords:** marine sponges, actinomycetes, *Streptomyces*, staphylococci, biofilms, contact lens

## Abstract

*Staphylococcus epidermidis* and *Staphylococcus aureus* are opportunistic pathogens that cause nosocomial and chronic biofilm-associated infections. Indwelling medical devices and contact lenses are ideal ecological niches for formation of staphylococcal biofilms. Bacteria within biofilms are known to display reduced susceptibilities to antimicrobials and are protected from the host immune system. High rates of acquired antibiotic resistances in staphylococci and other biofilm-forming bacteria further hamper treatment options and highlight the need for new anti-biofilm strategies. Here, we aimed to evaluate the potential of marine sponge-derived actinomycetes in inhibiting biofilm formation of several strains of *S. epidermidis, S. aureus*, and *Pseudomonas aeruginosa.* Results from *in vitro* biofilm-formation assays, as well as scanning electron and confocal microscopy, revealed that an organic extract derived from the marine sponge-associated bacterium *Streptomyces* sp. SBT343 significantly inhibited staphylococcal biofilm formation on polystyrene, glass and contact lens surfaces, without affecting bacterial growth. The extract also displayed similar antagonistic effects towards the biofilm formation of other *S. epidermidis* and *S. aureus* strains tested but had no inhibitory effects towards *Pseudomonas* biofilms. Interestingly the extract, at lower effective concentrations, did not exhibit cytotoxic effects on mouse fibroblast, macrophage and human corneal epithelial cell lines. Chemical analysis by High Resolution Fourier Transform Mass Spectrometry (HRMS) of the *Streptomyces* sp. SBT343 extract proportion revealed its chemical richness and complexity. Preliminary physico-chemical characterization of the extract highlighted the heat-stable and non-proteinaceous nature of the active component(s). The combined data suggest that the *Streptomyces* sp. SBT343 extract selectively inhibits staphylococcal biofilm formation without interfering with bacterial cell viability. Due to absence of cell toxicity, the extract might represent a good starting material to develop a future remedy to block staphylococcal biofilm formation on contact lenses and thereby to prevent intractable contact lens-mediated ocular infections.

## Introduction

Ocular devices such as intraocular lenses, posterior contact lenses, conjunctival plugs and orbital implants have aided in restoring and improving human vision. However, contamination of these devices with bacterial biofilms can lead to device-related ocular infections such as endophthalmitis, crystalline keratopathy, corneal ulceration, keratitis, lacrimal system, and periorbital infections (Bispo et al., 2015; Cho et al., 2015). The National Institute of Health (NIH) estimates that biofilms contribute to about 75% of the human microbial infections. Biofilms are surface-associated sessile microbial communities that are enmeshed in a self-produced extracellular matrix composed of polysaccharides, proteins, lipids and DNA (Flemming and Wingender, 2010; Oja et al., 2015). Compared to the free-living planktonic counterparts, bacteria in biofilms are 1000-fold more resistant to conventional antibiotic therapies and host immune responses (Donlan, 2001; Otto, 2009; Burmolle et al., 2010). The highly persistent and detrimental nature of biofilm-associated infections and rapid emergence of multidrug resistant strains (Barros et al., 2014; Sakimura et al., 2015) has imposed a major burden on health-care and medical settings. The current inexistence of effective biofilm-based therapeutics (Bjarnsholt et al., 2013) has necessitated the need for development of novel antibiofilm strategies for prophylaxis and/or treatment of the multitude of biofilm-associated ocular infections.

Staphylococci, particularly *S. epidermidis* and *S. aureus* are the most common causative agents of device-related infections. Infections caused by other staphylococci are far less frequent (Otto, 2009). *S. epidermidis* and *S. aureus* are commensal Gram positive bacteria found on human skin and nares, causing a wide range of indwelling medical device-related infections (Rogers et al., 2009; Uribe-Alvarez et al., 2016). The biofilm-based lifestyle of *S. epidermidis* and *S. aureus* on medical devices is a hallmark of the sub-acute and chronic recalcitrant infections caused by them (Reiter et al., 2014). Biofilm formation in *S. epidermidis* and *S. aureus* is facilitated predominantly by the synthesis of the homopolymer polysaccharide intercellular adhesion (PIA) by the enzymes coded by the *ica* locus. PIA-independent biofilm formation, mediated by surface proteins such as biofilm associated protein (Bap/Bhp) and accumulation associated protein (Aap), eDNA release, autolysins and cell sortase-anchored proteins have also been reported in several staphylococcal strains (Otto, 2009; Archer et al., 2011; McCarthy et al., 2015).

Marine actinomycetes represent an untapped reservoir of a broad range of biologically active compounds of pharmaceutical importance (Subramani and Aalbersberg, 2012; Zotchev, 2012; Lee et al., 2014; Azman et al., 2015). Particularly, the marine sponge-associated actinomycetes are well documented for their intrinsic chemical repertoire (Abdelmohsen et al., 2014a; Santos-Gandelman et al., 2014; Reimer et al., 2015). Novel secondary metabolites with discrete biological activities have been reported from sponge-associated actinomycetes (Abdelmohsen et al., 2014a; Grkovic et al., 2014; Cheng et al., 2015). These include antimicrobial (Hentschel et al., 2001; Eltamany et al., 2014), antiparasitic (Dashti et al., 2014; Viegelmann et al., 2014), immunomodulatory (Tabares et al., 2011), antichlamydial (Reimer et al., 2015), antioxidant (Abdelmohsen et al., 2012; Grkovic et al., 2014), anticancer (Vicente et al., 2015) and anti-biofilm (Oja et al., 2015; Park et al., 2016) activities. The extreme and dynamic conditions offered by the oceans (differences in temperature, pH, pressure, light intensities etc.) are the potential reasons often linked to the production of secondary metabolites by marine actinomycetes (Abdelmohsen et al., 2017). The frequent rediscovery of bioactive compounds and redundancy of sample strains from terrestrial environment has further made the marine actinomycetes as hotspots for discovery of new compounds (Dalisay et al., 2013). Among actinomycetes, the genus *Streptomyces* are considered to be the most prolific producers of secondary metabolites for medical, agriculture and veterinary usage (Tan et al., 2016). Over two-thirds of natural products isolated to date are from *Streptomyces* which indicates their huge biosynthetic potential and chemistry profiles. The rich genetic and metabolic diversity, and the ability to catabolize a wide range of compounds, has made the genus *Streptomyces* to be probed for discovery of novel compounds that could be translated to clinical applications (Hassan et al., 2016; Ser et al., 2016; Yang and Sun, 2016; Perez et al., 2016; Zhao et al., 2016).

In our continuing effort for discovery of anti-biofilm agents, we employed a crystal violet-based screening method to identify anti-biofilm activity of organic extracts generated from marine sponge-derived actinomycetes. The biofilm forming reference strain *S. epidermidis* RP62A was employed as a model for screening. Herein we report the inhibitory effects of an organic extract from marine sponge-associated *Streptomyces* sp. SBT343 against the biofilm formation of *S. epidermidis* RP62A on polystyrene, glass and contact lens surfaces. The potential anti-biofilm activity was tested on two other strains of *S. epidermidis*, four different *S. aureus* strains and two different *P. aeruginosa* strains. The results obtained highlighted that the extract exhibited potent anti-biofilm effects on all the staphylococcal strains tested but did not exert any effect on the *Pseudomonas* strains. Preliminary evaluations on the physico-chemical characterization of active component(s) in the extract suggested their heat stable and non-proteinaceous nature.

## Materials and Methods

### Pathogenic Strains and Growth Conditions

Bacterial strains used in the study are listed in **Table 1**. All strains for the biofilm study were propagated in Tryptic Soy Broth (TSB; Becton Dickinson) (17.0 g/l pancreatic digest of casein, 3.0 g/l papaic digest of soybean meal, 5.0 g/l sodium chloride, 2.5 g/l dipotassium hydrogen phosphate, 2.5 g/l glucose) and incubated at 37°C.

**Table 1:** Strains used in this study.

Strain	Description	Reference and/or source
*S. epidermidis* ATCC 12228	Non-infection associated strain	ATCC collection
*S. carnosus* TM300	Meat starter culture	Rosenstein et al., 2009
*S. epidermidis* RP62A	Reference strain isolated from intra-vascular catheter associated sepsis	ATCC collection
*S. epidermidis* O-47	Clinical isolate from septic arthritis	Heilmann et al., 1996
*S. epidermidis* 1457	Clinical isolate from a patient with infected central venous catheter	Mack et al., 1992
*S. aureus* SH1000	MSSA; *rsbU* derivative of 8325-4 *rsbU^+^*	Horsburgh et al., 2002
*S. aureus* RN4220	Restriction-deficient transformation recipient; originally derived from NCTC 8325-4	Kreiswirth et al., 1983
*S. aureus* Newman	MSSA isolate from osteomyelitis patient	Lipinski et al., 1967
*S. aureus* USA300	CA-MRSA isolate from a wrist abscess	McDougal et al., 2003
*P. aeruginosa* PAO1	Clinical isolate from wound	Dr. Vinay Pawar, Braunschweig, Germany
*P. aeruginosa* PA14	Clinical isolate from burn wound	Dr. Vinay Pawar, Braunschweig, Germany

### Fermentation Conditions and Extract Preperation

*Streptomyces* sp. SBT343 was cultivated from the Mediterranean sponge *Petrosia ficiformis* that was collected offshore Pollonia, Milos, Greece (N36.76612°; E24.51530°) in May 2013 (Cheng et al., 2015). Briefly, 1% (v/v) inoculum of a well grown culture of *Streptomyces* sp. SBT343 was inoculated in 150 mL modified ISP2 medium (2.5 g/l malt extract, 1.0 g/l yeast extract, D-mannitol 25 mM, in artificial sea water) in a 250 mL conical flask and the strain was subjected to batch fermentation (incubation at 30°C with shaking at 150 rpm) for 10 days. After harvesting, the filtrate of the fermented culture was extracted twice with 250 mL ethyl acetate for each time. The extract was generated by evaporating the solvent in a rotational evaporator (Heidolph Laborota, 4001, Büchi Vacuum Controller V-805). The modified ISP2 broth medium control ensured the purity of the fermentation and was also extracted separately in a similar manner and this served as the medium control for the bioactivity testing. Extracts were dissolved in DMSO (final concentration 3.75% on the cells) and used for anti-biofilm assays.

### Quantification of Biofilm Formation

Quantification of biofilm formation was performed according to Weisser et al. (Weisser et al., 2010). Briefly, overnight culture of bacterial strains were diluted with TSB medium to OD_600_ of 0.05, and incubated statically in a 96-well flat bottom polystyrene plate (Greiner bio-one, GmbH, Germany) with or without the varying concentrations of the *Streptomyces* sp. SBT343 extract (0, 31.25, 62.5, 125, and 250 μg/ml) at 37°C (for *S. epidermidis* and *P. aeruginosa*) or 30°C (for *S.* aureus) for 24 h. Extract from the modified ISP2 medium control (250 μg/ml) served as the control for the experiment. For biofilm quantification, planktonic bacteria were discarded, and the wells were rinsed carefully with sterile 1X phosphate buffered saline (PBS) twice, and the adherent cells in the plate were heat-fixed at 65°C for 1 h. This was followed by staining with 0.3% crystal violet for 5 min. The stained biofilm was washed with sterile water thrice. Plates were dried in an inverted position and OD_492_ readings were measured to compare the extent of biofilm inhibition in the extract treated sets vs. the modified ISP2 medium control treated set. The biofilm-negative *S. epidermidis* (ATCC12228) and *S. carnosus* TM300 served as the negative controls in the experiment.

### Effect of the Extract on Existing Biofilms

To study the effect of the *Streptomyces* sp. SBT343 extract on the existing biofilm, biofilms were established by incubating the bacteria (inoculation OD_600_ 0.05) in TSB medium in a 96-well flat bottom polystyrene plate at 37°C or 30°C for 24 h. Planktonic bacteria were discarded by inverting the plate on paper stacks. This was followed by addition of fresh TSB medium with varying concentration of the *Streptomyces* sp. SBT343 extract and the plate was again incubated statically at 37°C or 30°C for 24 h. The effect of the extract in eradication of preformed biofilm by the extract was measured by the crystal violet assay as explained above. Extract from the modified ISP2 medium control (250 μg/ml) and sodium metaperiodate (40 mM) served as the negative and positive controls in the experiment.

### Growth Measurements

The effect of *Streptomyces* sp. SBT343 extract on the growth of staphylococcal strains was evaluated (Nithya et al., 2010) by growth curve analysis. The growth curves were determined up to 24 h. The extract at 50% biofilm inhibitiory concentration (BIC_50_) and the highest tested concentration (125 μg/ml) (non-toxic to cell lines), was added to a conical flask containing bacteria at an OD_600_ of 0.1. The flasks were incubated 37°C under shaking conditions at 200 rpm. TSB medium without the bacteria served as negative control. Growth medium with the modified ISP2 medium extract (125 μg/ml) and bacteria served as the control. The reading was observed continuously up to 24 h at 2 h intervals. The experiment was carried out with three independent cultures.

### Investigation on the Appearance of Biofilm-Switches Upon Extract Treatment

In order to study the appearance of biofilm-switches [PIA-dependent to PIA-independent (protein-mediated) biofilms] in the presence of the extract, the residual biofilms after the extract treatments were challenged with metaperiodate (40 mM NaIO_4_) for PIA-dependent and proteinase K (1 mg/ml in 100 mM Tris-Cl) for PIA-independent (protein-mediated) modes of biofilm formation with the procedure described elsewhere (Wang et al., 2004).

### Scanning Electron Microscopy

In order to evaluate the effect of the extract on the biofilm formation on soft contact lens (Proclear^®^, CooperVision^®^ Lensbest, Germany), round pieces (diameter of 6 mm) were punched out of each contact lens using a sterile puncher. Each piece was washed twice with sterile 1XPBS and placed in a 24 well plate (Greiner bio-one, GmbH, Germany) containing 1 mL of *S. epidermidis* RP62A (OD_600_ 0.05) in TSB with the extract (at concentrations of 62.5, 125, and 250 μg/ml). For testing the effect of the extract on the biofilm formation on glass surface, sterile glass cover slips (diameter of 12 mm) were placed in a 24 well plate containing 1 mL *S. epidermidis* RP62A (OD_600_ 0.05) in TSB with the extract (at concentrations of 62.5, 125, and 250 μg/ml). In each case, bacteria treated with extract (250 μg/ml) from modified ISP2 medium served as the control, while wells with the TSB and the contact lens or cover slips alone served as sterile controls, respectively. Plates containing the contact lens and cover slips sets were then incubated statically at 37°C for 24 h. Samples were then washed carefully with sterile 1XPBS twice, and fixed overnight with 6.25% gluteraldehyde (in 50 mM phosphate buffer pH 7.4). After overnight fixation, samples were washed 5 times with Sörenson buffer (100 mM KH_2_PO_4_ and 100 mM Na_2_HPO_4_) and transferred to the electron microscopy unit, where they were dehydrated and then coated with gold by a low vacuum sputter coating, and scanned by scanning electron microscopy.

### Confocal Microscopy

Samples on the contact lens and the cover slips were prepared and treated as above. After overnight incubation in 24 well plates, samples were subjected to a rapid epifluoroscence staining method employing the bacterial viability kit Live/Dead Bac Light, Invitrogen Ltd., Paisley, UK. The kit employs Syto 9 green and propidium iodide red fluorescent nucleic acid stains for distinguishing live and dead cells, respectively. The dye was prepared and added to the samples according to the manufacturer’s specifications. Samples with the dye were incubated in the dark for 15 minutes and then assessed by a confocal microscope. The final step comprised of acquiring photographs of the samples with a Leica Microystems (Leica TCS SP5, Leica Microsystems, Germany) at excitation levels of 488 nm for Syto 9 and 543 nm for propidium iodide. Images were acquired at 1.5 μm intervals. Further, images representing two dimensional [compressed *z* series (*x–y* sides) and compressed (*x–z*) views] and three dimensional views of the biofilm were acquired with IMARIS v 8.1.2. The thickness of the biofilms was also calculated with IMARIS v 8.1.2.

### Cytotoxicity Studies

Human Epithelial Corneal Cells (HCEC) were cultivated in DMEM/Ham’s F12 (Invitrogen Life Technologies, USA), supplemented with 5% (v/v) FBS, 1% (w/v) L-glutamine, 0.4% (w/v) antibiotics (50 U/ml penicillin and 50 mg/ml streptomycin), insulin (5 mg/l) and EGF (10 μg/l) (PAA Laboratories GmbH, Austria). To assess the cytotoxicity on HCEC, vitality assay was performed with slight modifications (Schmitt et al., 2002). Briefly, vitality staining was performed with different concentrations of SBT343 extract for 24 h. 3.5 × 10^5^ cells were seeded in 6-well plates for 24 h in a control medium. After treatment, cells were collected, and 70 μl of the cell suspension was stained with 30 μl staining solution [Gel RedBiotrend (Köln, Germany) and fluorescein diacetate (Kreiswirth et al., 1983)]. Twenty microliter of this mixture was applied to the slide, and the fractions of green and red cells in a total of 200 cells were counted at a 500-fold magnification with a fluorescence microscope. Macrophage (J774.1) and mouse fibroblast (NIH/3T3) cell lines were cultured in RPMI 1640 (1X)+Glutamax^TM^-I and DMEM (1X)+Glutamax^TM^-I (Life Technologies^TM^,USA) supplemented with 10%FCS, without antibiotics. Cytotoxicity on J774.1 and NIH/3T3 was assesssed employing alamar blue assay (Huber and Koella, 1993). 1 × 10^5^ cells/ml were seeded in 96-well plates containing the extract at different concentrations (ranging from 31.25 to 500 μg/ml) and were incubated for 24 h at 37°C with 5% CO_2._ After incubation, 20 μL alamar blue (Thermofischer scientific, USA) was added to each well and the plates were further incubated for 24 h at 37°C with 5% CO_2._ Finally, the OD_550_ values of the plates were measured and normalized with OD_630_ values. The extent of cytoxicity was determined by comparing the extract treated sets with the control. The final DMSO concentration on the cells was 1%.

### Physico-chemical Characterization of Anti-biofilm Component(s)

To understand the nature of the active component(s), the extract was subjected to thermal and enzymatic (proteinase K and trypsin) treatments. Briefly, the extract was subjected to heat treatments at 50, 75, and 100°C for 1 h and cooled on ice. For the enzymatic treatment, proteinase K or trypsin (at a final concentration of 1 mg/ml) was added to the extract (at a final concentration of 0.125 mg/ml) and the reactions were incubated for 1 h at 37°C. As controls, extracts were incubated for 1 h at 37°C without the enzymes, a treatment that did not impair the anti-biofilm effect. For each of the above tests, the biofilm inhibitory effects of treated and untreated extracts were compared using the microtitre plate assay (for biofilm formation) against all the staphylococcal strains tested. Each data point is composed of three independent experiments performed in quadruplicate.

In parallel, the activity of proteinase K and trypsin (1 mg/ml each) in the presence of DMSO were independently assessed employing the quantitative azocasein assay (Hasegawa et al., 2008).

### Quantitative RT-PCR

After complementation of *S. epidermidis* RP62A with SBT343 extract at 62.5 (BIC_50_) and 250 μg/ml for 24 h at 37°C, total RNA was isolated from planktonic bacteria and those in biofilm employing Trizol reagent (Invitrogen, Paisley, UK) and FastPrep^®^ disrupter (Thermo Savant, Qbiogene, Inc., Cedex, France). Firstly, 1 ml of cells (from planktonic and biofilm states; the biofilm was gently removed from 24 well plate using a sterile scraper and re-suspended in 1 ml of fresh TSB medium) were centrifuged at 13000 rpm for 10 min at 4°C and pellets were re-suspended in 1 ml Trizol reagent. This suspension was briefly homogenized in a Lysing Matrix E tube (MP Biomedicals Germany, GmbH, Eschwege, Germany) in the FastPrep^®^ cell disrupter and subjected to chloroform-based RNA extraction method. Purity and concentration of the extracted total RNA was spectrophotometrically assessed using a NanoDrop ND-1000 (peqLab Biotechnologie, GmbH). The A_260_/A_280_ values of (range 1.8–2.0) indicated the purity of the RNA samples and the mean RNA yield obtained was 182.63 μg/ml. All the RNA samples were digested with DNase I (Thermo Scientific). Briefly, 1 μg of RNA was digested with 1 μl of DNase by incubation at 37°C for 30 min. This was followed by addition of 1.5 μl of DNase stop solution (50 mM EDTA) and incubation at 70°C for 10 min. After quality assessment of RNA after DNase treatment, about 50 ng/μl of RNA was used as template for qPCR experiment.

cDNA synthesis and qPCR amplification was performed simultaneously by employing the power SYBR^®^ Green RNA-to-C_T_^TM^ 1-step kit (Applied Biosystems, GmbH, Germany). Primers used in the study were designed according to the literature (Reiter et al., 2014), and were commercially produced (Eurofins MWG Synthesis GmbH, Germany). These primers were chosen based on their thermodynamic and sequence parameters. The reaction mixture for qPCR contained 1 μl of RNA template, 5 μl of power SYBR^®^ Green RTPCR mix, 1 μl each of forward and reverse primers (10 μM), 0.08 μl of reverse transcriptase provided by the manufacturer and 1.92 μl of RNase free water. qPCR was performed using the Bio-Rad C1000 Touch^TM^ thermal cycler with the following cycle parameters: holding stage of 48°C for 30 min and 95° C for 10 min, followed by 50 cycles of 95°C for 15 s and then 55°C for 1 min, with a final melting curve determination. Experiment was peformed with three technical and biological replicates each. A difference of ≥7 Ct (cycle threshold) between the cDNA sample and no-template PCR control was considered negligible for relative quantification analysis. Finally, the relative expression of the target genes (*IcaA* and *IcaR*) in the presence of SBT343 extract in relation to the modified ISP2 extract treated control was determined using the comparison with the expression level of the reference gene, DHFR (the expression of DHFR gene stayed constant in the conditions tested). The significance of the relative quantification was assessed by Student’s *t*-test (GraphPad Prism^®^ version 6.01).

### Statistical Analysis

Experiments were repeated at least three times in quadruplicates and the data were expressed as mean ± standard error mean. The Student’s *t*-test was used and *p* < 0.05 was considered as statistically significant. GraphPad Prism^®^ version 6.01 was used for statistical analysis of the experimental data.

### LC-MS Analysis

Analytical grade reagents, Methanol (MeOH), dichloromethane (DCM), acetonitrile (MeCN), and formic acid were purchased (Fisher Scientific, Hemel Hempstead, UK). In-house HPLC grade water was used from a direct Q-3 water purification system (Millipore, Watford, UK). Samples and medium control samples were prepared at a concentration of 1 mg/mL in 80:20 MeOH: DCM with a solvent blank. Experiments were performed with an Exactive mass spectrometer with an electrospray ionization source attached to an Accela 600 HPLC pump with Accela autosampler and UV/Vis detector (Thermo Scientific, Bremen, Germany). The mass accuracy was set to less than 3.0 ppm. The Orbitrap mass analyzer can limit the mass error within ±3.0 ppm. The instrument was calibrated to maintain a mass accuracy of ±1.0 ppm by applying the lock mass function. The instrument was externally calibrated per the manufacturer’s instructions before the run and was internally calibrated during the run using lock masses. Mass spectrometry was carried out over a mass range of 100–2000 *m*/*z* in positive and negative ionization modes with spray voltage of 4.5 kV and capillary temperature at 270°C. Ten microliter were injected from each vial, at a flow rate of 300 μL/min. The column used was an ACE5 C18 column (5 μm × 75 mm × 3 mm) (Hichrom Limited, Reading, UK). A binary gradient method was utilized. The two solvents were A (water and 0.1% formic acid) and B (MeCN and 0.1% formic acid). The gradient was carried out for 10 min and the program followed; at zero minutes A = 90% and B = 10%, at 30 min A = 0% and B = 100% at 36 min A = 90% and B = 10% until end at 45 min. The UV absorption wavelength was set at 254 nm, the sample tray temperature was maintained at 4°C and the column maintained at 20°C. The samples were run sequentially, with solvent and media blanks analyzed first. LC-MS data was acquired using Xcalibur version 2.2 (Thermo Scientific, Bremen, Germany). LC-MS chromatograms were subsequently obtained using MassLynx v 4.10. This was followed by dereplication strategy. Since, the modified ISP2 medium is a complex mixture of constituents and could interfere with the identification of secondary metabolites in the SBT343 extract, a medium blank was analyzed together with the bacterial extract and obtained features were regarded as interference and subtracted for detection of true bacterial secondary metabolites. Finally, the *m/z* values were searched for possible hits in the MarinLit^®^ database (Abdelmohsen et al., 2014b; Macintyre et al., 2014).

## Results

### Anti-biofilm Effect of the *Streptomyces* sp. SBT343 Organic Extract

**FIGURE 1:**
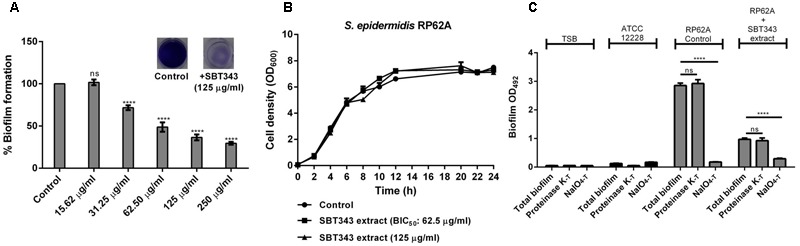
**(A)** Dose dependent inhibition of *Streptomyces* sp. SBT343 extract on biofilm formation of *S. epidermidis* RP62A on polystyrene flat-bottom 96-well plates. *S. epidermidis* RP62A treated with the extract from modified ISP2 medium (250 μg/ml) served as the appropriate control in the experiment. **(B)** Growth curve of *S. epidermidis* RP62A in the presence of BIC_50_ (62.5 μg/ml) and highest tested concentration (125 μg/ml) of the SBT343 extract. **(C)** Investigation of the *Streptomyces* sp. SBT343 for induction of PIA-independent biofilm formation in *S. epidermidis* RP62A. Control and the residual biofilms after the extract treatment (250 μg/ml) were subjected to two parallel treatments (i) with metaperiodate (NaIO_4-T_), which is suitable to dissolve the PIA-matrix proportion of the biofilm, and (ii) with proteinase K (proteinase K_-T_) which will reduce proteins engaged in the biofilm matrix. Biofilm measurements were done shortly after the respective treatments, and the total biofilm levels were compared with the untreated control and residual biofilm sets. The biofilm-negative *S. epidermidis* ATCC12228 served as the negative control. *S. epidermidis* RP62A treated with the extract from modified ISP2 medium (250 μg/ml) served as the appropriate control. Graphs represent mean values ± SEM from three independent repetitions of the experiment done in quadruplicate. ns, not significant, ^∗∗∗∗^*p* < 0.0001.

Our continuing effort for discovery of anti-biofilm agents from marine sponge-derived actinomycetes against the model isolate *S. epidermidis* RP62A, led to identification of the anti-biofilm *Streptomyces* sp. SBT343 extract. The presence of the extract at 31.25 μg/ml during the bacterial growth caused a significant (*p* < 0.0001) reduction in the biofilm formation after 24 h of growth. At 62.5 μg/ml about 50% of the biofilm formation was inhibited and this was designated as the Biofilm Inhibitory Concentration (BIC_50_). The anti-biofilm activity of the extract was dose-dependent, leading to 71.35% inhibition of biofilm formation at the maximum concentration (250 μg/ml) tested (**Figure 1A**). Even after the addition of extract at BIC_50_ and highest tested concentration, the growth of *S. epidermidis* RP62A was at the same level as that of the control. These results confirm that the biofilm inhibition by the extract is not due to growth effect (**Figure 1B**). SBT343 extract showed no effect on detaching/dispersing the bacteria from preformed biofilm at any of the tested concentrations (data not shown).

As the strain *S. epidermidis* RP62A is known as a strong PIA matrix biofilm producer, SBT343-mediated biofilm inhibition strongly suggests interference with PIA-mediated biofilm formation. Since, staphylococci are known to switch from PIA-dependent to PIA-independent biofilm formation under different conditions; the presence of these spontaneous switches in the presence of the extract was assessed. The ineffectiveness of proteinase K treatment on residual biofilms highlight that there is no spontaneous switch from PIA-dependent to PIA-independent (protein-mediated) biofilm formation in the presence of SBT343 extract (**Figure 1C**).

### SEM Analysis

**FIGURE 2:**
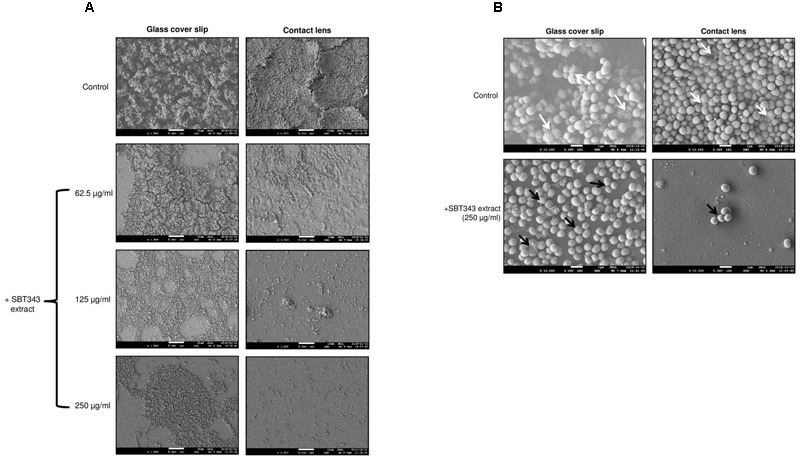
**Representative SEM images of staphylococcal biofilm on (A)** Glass cover slip and contact lens at X1500 magnification. **(B)** Glass cover slip and contact lens at X10000 magnification. The white arrows indicate the presence of fibrous, net like structures which are typical features of PIA-dependent biofilms and the black arrows indicate the absence of the same. *S. epidermidis* RP62A treated with the extract from modified ISP2 medium (250 μg/ml) served as the appropriate control in the microscopy experiments.

The biofilm inhibition potential of the SBT343 extract was studied on glass and contact lens surfaces using microscopic techniques. Electron microscopy of biofilm formation on glass cover slips and contact lenses in the presence of 62.5, 125, and 250 μg/ml SBT343 extract confirmed the results obtained by the *in vitro* crystal violet assay (**Figure 2**). In the control glass cover slips, mushroom shaped, three-dimensional biofilm was observed, whereas, multi-layered biofilm was observed on control contact lenses. In the glass cover slips and contact lenses incubated with the SBT 343 extract (62.5, 125, and 250 μg/ml), a dose-dependent reduction in the biofilm was clearly seen (**Figure 2A**). The reduction of biofilm with the extract was even pronounced in the contact lens model suggesting its possible application in contact lens solution and storage systems. Surfaces were distinctly seen between sporadic microcolonies in the glass cover slip and the contact lens incubated with higher concentrations of the extract. Representative SEM images at higher magnification indicated that the extract did not affect the morphology of the staphylococcal cells. Further, the presence of fibrous, net-like structures in the biofilm matrix was greatly reduced in both the glass cover slips and contact lenses incubated with SBT343 extract (**Figure 2B**). These findings suggest that the extract possibly works by altering the biofilm matrix composition or interferes with the production of extracellular matrix.

### Confocal Microscopy Analysis

**FIGURE 3:**
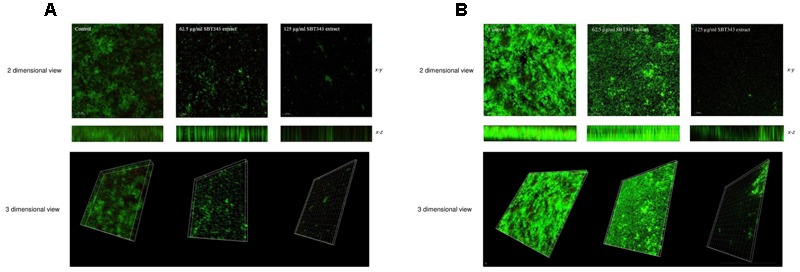
**Confocal laser scanning microscopy analyses of staphylococcal biofilm in the presence of the SBT343 extract, visualized by fluorescence vital dye.** Images were acquired by Leica TCS SP5 with a 20X objective lens (scale bar 30 μm). **(A)** Biofilm images on the glass cover slip. **(B)** Biofilm images on contact lens. In **(A,B)**, compressed *z* series (top), where multiple *x–y* planes from top to bottom of the biofilm are combined and the smaller image (bottom) represents the compressed *x–z* (side) of the biofilm. The thickness of the biofilms in control was 28.7 μm, whereas after extract treatment it was only 15.1 μm (62.5 μg/ml) and 4.53 μm (125 μg/ml) on the glass cover slips. Similarly, the thickness of the biofilms in the extract treated contact lens samples was only 13.6 μm (62.5 μg/ml) and 7.55 μm (125 μg/ml).

For the confocal microscopy studies, effect of the SBT343 extract on the biofilm formation was also examined with two systems, first in the cover slips, where the control sample showed compact and condensed biofilm, while the treatment with the extract attenuated the formation of the biofilm in a dose dependent matter. The same observations were obtained in the contact lens as a second system for examination. The ability of the extract to inhibit the biofilm formation was stronger in the coverslips experiments in comparison to the contact lens, but in both systems the inhibition was significant in comparison to the control (**Figure 3**). Noteworthy, very few non-viable bacteria (stained red) were spotted in the experiment both in the treated and the untreated sets. This further confirms that the extract does not interfere with bacterial cell viability. As a control, cells in biofilm were exposed to 75% ethanol for 5 mins and stained with propidium iodide and SYTO green. In this case a large population of dead cells were spotted in the microscope (data not shown). Images representing compressed *x–y* and *x–z* (side) views (**Figure 3**) indicated that the number of the bacteria in the biofilm and the total biofilm thickness in the extract treated cover slips and contact lens sets were greatly reduced in comparison with the compact and condensed biofilm control. These images further corroborated the results that the reduction in fluorescence in the presence of the extract, was primarily due to the repression of biofilm formation, and has no negative effects on bacterial cell viability.

### Anti-biofilm Effect of SBT343 Extract on Other Pathogens

**FIGURE 4:**
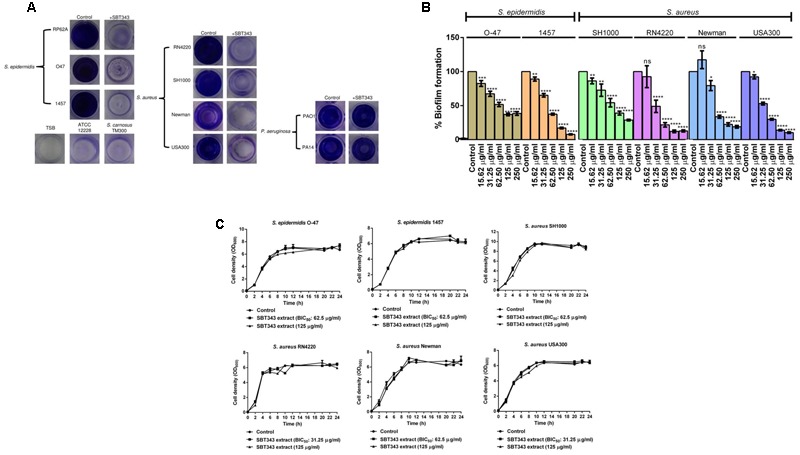
**Anti-biofilm effect of SBT343 extract on other staphylococcal strains. (A)** Photographs of wells of 96-well plate showing biofilms in the absence and presence of SBT343 extract after crystal violet staining. Control (modified ISP2 medium extract: 125 μg/ml) and SBT343 extract treated (125 μg/ml). **(B)** Dose dependent inhibitory effect of *Streptomyces* sp. SBT343 extract on biofilm formation of other staphylococcal strains on polystyrene flat-bottom 96-well plates. Staphylococcal strains treated with the extract from modified ISP2 medium (250 μg/ml) served as the appropriate control in the experiment; **(C)** Growth curves of staphylococcal strains in the presence of BIC_50_ and highest tested concentration (125 μg/ml) of the SBT343 extract. Graphs represent mean values ± SEM from three independent repetitions of the experiment done in quadruplicate. ns: not significant, ^∗^*p* < 0.05, ^∗∗^*p* < 0.01, ^∗∗∗^*p* < 0.001, and ^∗∗∗∗^*p* < 0.0001.

The anti-biofilm activity of the SBT343 extract was investigated with other biofilm-forming *S. epidermidis, S. aureus*, and *P. aeruginosa* strains (**Table 2**). SBT343 extract (125 μg/ml) significantly reduced the biofilm formation of all the *S. epidermidis*, MSSA and MRSA strains tested, while the biofilm formation of *P. aeruginosa* was unaffected (**Figure 4A**). A dose dependent reduction in biofilm formation of the staphylococcal strains was observed upon extract treatment (**Figure 4B**), while the growth of the strains were unaltered in the presence of the extract (**Figure 4C**). Further, the extract had no effects on the preformed biofilms of all the strains tested at the highest concentration tested (data not shown). This indicates selectivity of the extract in inhibiting staphylococcal biofilms.

**Table 2:** Biofilm formation of the investigated bacterial strains employing crystal violet assay.

Strain	Biofilm (OD 492 nm)
*S. epidermidis* ATCC 12228	0.124 ± 0.010
*S. carnosus* TM300	0.204 ± 0.013
*S. epidermidis* RP62A	2.861 ± .0.143
*S. epidermidis* O-47	1.387 ± 0.044
*S. epidermidis* 1457	2.165 ± 0.069
*S. aureus* SH1000	0.994 ± 0.112
*S. aureus* RN4220	1.177 ± 0.092
*S. aureus* Newman	0.691 ± 0.149
*S. aureus* USA300	0.659 ± 0.031
*P. aeruginosa* PAO1	1.337 ± 0.101
*P. aeruginosa* PA14	1.331 ± 0.067

*Each data point is composed of three independent experiments performed in quadruplicate. Standard errors are reported.*

### Cytotoxicity Analysis

We further investigated the cytotoxicity profile of the SBT343 extract against mouse fibroblast (NIH/3T3), macrophage (J774.1) and human corneal epithelial cells (HCEC). Results from vitality test and alamar blue assay demonstrated that cells did not suffer from significant toxicity after 24 h with effective concentrations of the extract (in the range of 31.25–125 μg/ml). The highest concentration 500 μg/ml displayed moderate to high cytotoxic effects on the cell lines tested (**Table 3**).

**Table 3:** Cytotoxic evaluation for SBT343 extract.

Cell line	% reduction in cell viability		
	500 μg/ml	250 μg/ml	31.25–125 μg/ml
HCEC	90.75 ± 1.23^∗∗∗∗^	20.66 ± 5.10^∗^	NC
NIH/3T3	21.56 ± 2.43^∗∗∗∗^	NC	NC
J774.1	33.83 ± 2.27^∗∗∗∗^	NC	NC

*Each data point is composed of three independent experiments performed in quadruplicate. Standard errors are reported. Differences in mean were compared to untreated control and considered statistically significant when p (^∗^*p* < 0.05, ^∗∗∗∗^*p* < 0.0001) as per Student’s *t*-test. NC, not cytotoxic.*

### Physico-chemical Characterization of Anti-biofilm Component(s)

**FIGURE 5:**
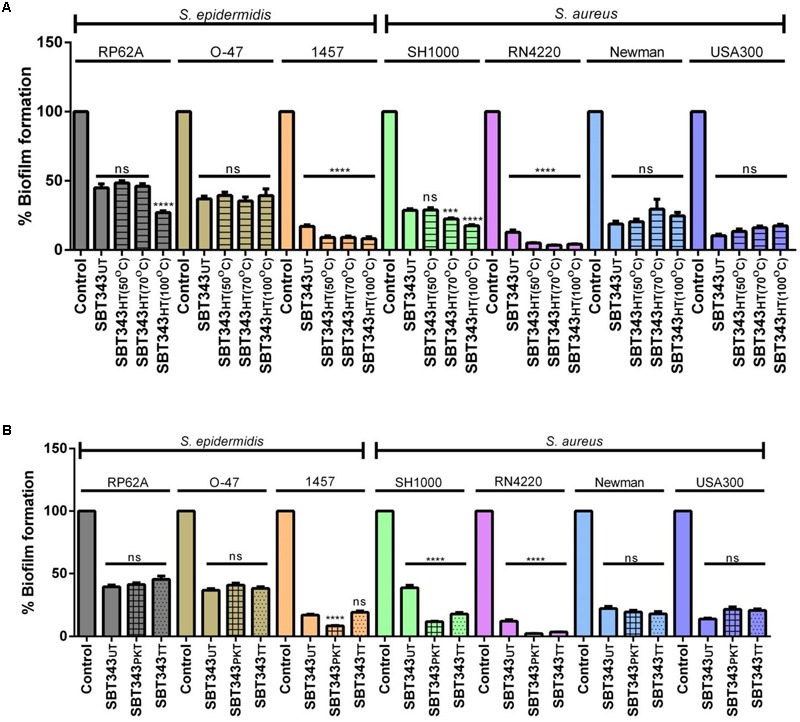
**Physico-chemical characterization of anti-biofilm component(s). (A)** Influence of heat treatment on anti-biofilm activity. UT, untreated; HT, heat treated. **(B)** Influence of enzyme treatment on anti-biofilm activity. UT, untreated; PKT, proteinase K treated; TT, trypsin treated. ns, not significant; ^∗∗∗∗^*p* < 0.0001.

Preliminary physical and chemical characterization of the biofilm inhibiting component(s) in the extract was assessed by subjecting the extract to physical (heat) and chemical (proteinase K or trypsin) treatments. Both the physical and chemical treatments, did not reduce the activity of the extract against all the staphylococcal strains (**Figure 5**). However, a slight increase in the activity was observed upon heat treatment, in *S. epidermidis* RP62A and 1457, and *S. aureus* SH1000 and RN4220 strains. Similarly, a slight increase in the anti-biofilm activity was observed upon enzyme treatment in *S. aureus* SH1000 and RN4220 strains (**Figure 5**). This suggests that the active component(s) in the extract is thermo-stable and non-proteinaceous in nature, and the extract contains compound(s) with anti-biofilm activity that could work similarly on different staphylococcal strains. DMSO at the tested concentrations did not influence proteinase K and trypsin activities (Supplementary Figure 1).

### LC-MS Analysis

**FIGURE 6:**
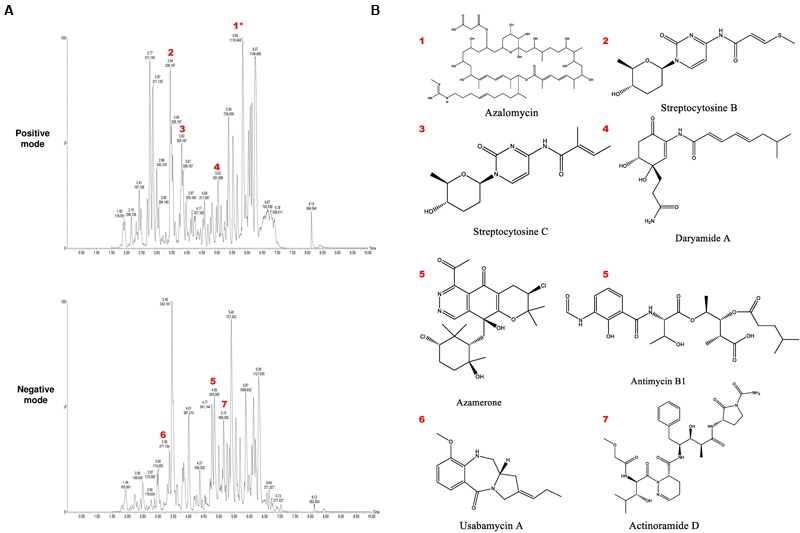
**(A)** Total ion chromatogram of crude extract of *Streptomyces* sp. SBT343 in both positive and negative modes, annotated to indicate metabolites identified in **Table 4**. Annotation and dereplication was done by eliminating the metabolites from the extract of modified ISP2 medium. Those annotated with an asterisk ^∗^ are sodium ion adducts. **(B)** Structures of the putatively identified and dereplicated compounds.

To identify the putative bioactive secondary metabolites in the SBT343 extract, the LC-UV/MS signature of the extract generated by high resolution Fourier Transform mass spectrometry was compared with the MarinLit database (a database for marine natural products). The dereplication of the chemical profile of SBT343 extract by comparison of HRMS data with MarinLit, led to identification of several known and unknown metabolites which were previously isolated from the genus *Streptomyces*. Both the positive and negative modes of electrospray ionization spectral data were used for dereplication purposes. The total ion chromatogram of the SBT343 extract showing the distribution of known and unknown compounds is depicted in **Figure 6A**. Further, the known compounds detected from the dereplication strategy are mentioned in **Table 4** and their structures are depicted in **Figure 6B**.

**Table 4:** Putatively identified and dereplicated compounds from the high-resolution mass spectral data sets of the crude ethyl acetate extract of *Streptomyces* sp. SBT343 using MarinLit^®^ database with a precision of ±0.1–1.0.

Peak ID	ESI Mode	*m/z*^∗^	Rt (min)	Hits (*m/z*^∗∗^)
1	P	1095.624	5.85	Azalomycin (1095.682)
2	P	325.197	3.44	Streptcytosine B (325.109)
3	P	307.187	3.83	Streptcytosine C (307.153)
4	P	350.098	5.03	Daryamide A (350.184)
**5**	N	510.265	4.85	Azamerone (510.168)
				Antimycin B1 (510.221)
**6**	N	272.154	3.38	Usabamycin A (272.152)
**7**	N	659.355	5.15	Actinoramide D (660.738)

*P, positive ESI mode; N, negative ESI mode; Rt, retention time; ^∗^ indicates the neutral *m/z* values of peaks found in the crude ethyl acetate extract of *Streptomyces* sp. SBT343; ^∗∗^ indicates the *m/z* values of the corresponding hits identified with the MarinLit^®^ database.*

## Discussion

Biomaterials used in clinical and medical settings are ideal niches for formation of microbial biofilms (Shanmughapriya et al., 2012). Even though a number of natural and synthetic anti-biofilm agents have already been discovered (Richards and Melander, 2009; Gupta et al., 2016; Miquel et al., 2016; Tran et al., 2016), none of them has entered the market, owing to obstacles in translational research and lack of interest by pharmaceutical and biomedical companies (Romero and Kolter, 2011). Hence, there is a large unmet need for development of anti-biofilm formulations to tackle the problem of biofilms.

Various culture dependent and independent techniques have revealed the bacterial biofilm diversity in contact lens-related corneal diseases (Hall and Jones, 2010; Wiley et al., 2012). Staphylococci, particularly *S. epidermidis*, are frequent contaminants of a range of ocular devices. It was estimated by Hou et al. (2012) that many ophthalmic isolates of *S. epidermidis* could form biofilms *in vitro*. Strong biofilm formation by *S. epidermidis* has been observed on various intraocular and contact lens (IOLs) materials (Okajima et al., 2006). Bacteria in the biofilms of contact lenses are frequently resistant to antimicrobials in the soft contact lens care products (Szczotka-Flynn et al., 2009). This has sharpened the need to develop new anti-biofilm-based contact lens products for combating ocular infections. The anti-biofilm potential of natural product based preparations, extracts, and compounds have increasingly been reported in *in vitro* biomaterial-based models (Pacheco da Rosa et al., 2013; Nair et al., 2016). Several researchers have tried to assess the anti-biofilm aspects of marine bacteria (Nithya et al., 2011; Gowrishankar et al., 2012; Papa et al., 2015; Wu et al., 2016), particularly, marine actinomycetes (Oja et al., 2015; Saleem et al., 2015; Park et al., 2016).

Leshem et al. (2011) and Cho et al. (2015) have previously reported the inhibitory effect of natural product based extract and several compounds on staphylococcal biofilm formation on contact lens and contact lens cases. We have shown that the organic extract from marine *Streptomyces* sp. SBT343 exhibits similar anti-biofilm effects at much lower concentrations (62.5–250 μg/ml). The extract had no adverse effects on the contact lens material at the highest concentrations tested (data not shown). At the same time, the lowest effective concentration of the extract did not show apparent cytotoxic effects on the three different cell lines tested which indicates the possibility of using the extract for the human subjects. The selective anti-biofilm effect of the SBT343 extract against different staphylococcal strains without interference with the bacterial cell growth suggests the less possible appearance of resistant mutants with the usage of this extract. However, further experiments are needed to prove this hypothesis. The basis of employing TSB medium as the growth medium in the study is to provide optimal growth conditions at which the effects of SBT343 extract could be determined in short periods.

Under certain conditions, *S. epidermidis* is known to switch between the PIA-dependent and independent modes of biofilm lifestyle (Rohde et al., 2005; Hennig et al., 2007). Our findings suggest that there are no switches in the biofilm lifestyle of the organism in the presence of the extract (**Figure 1C**). For a better understanding of the mechanism of biofilm inhibition by the extract, the relative mRNA expression of *icaA* and *icaR* in *S. epidermidis* RP62A (after 12 and 24 h) was determined in the planktonic and the biofilm cells in the presence of the extract. However, no significant changes in the expression levels of *icaA* and *icaR* were noted in the extract treated planktonic and biofilm cells (data not shown). These results indicate that the extract possibly works by an alternate mechanism and a global gene expression analysis would assist in deciphering the exact mode of action of the extract. The universal anti-biofilm activity against different staphylococcal strains with no effects on preformed biofilm and low cytotoxicity of this extract suggests its potential usage in contact lenses storage cases to prevent contact lens-associated ocular infections. Further studies are necessary to determine the anti-biofilm effect of SBT343 extract on different contact lens and storage cases materials.

The preliminary physical and chemical characterization of the anti-biofilm component(s) in the extract indicates that the active component(s) is of thermo-stable and non-proteinaceous nature and could act similarly on different staphylococcal strains tested. Dereplication strategies are often used in the natural products-based research for rapid identification of secondary metabolites in the crude bacterial extracts (Abdelmohsen et al., 2014b; Cheng et al., 2015). Currently, several analytical methods and tools are available for dereplication of metabolites in complex mixtures. Comparison of the HRMS data at positive and negative modes with the MarinLit database resulted in identification of several putative compounds in the *Streptomyces* sp. SBT343 extract. It is clear from the chromatogram that several peaks were not identified by comparison to the database, including some of the major components that showed strong peak intensity and good resolution. The high number of unidentified compounds highlights the chemical potential of this strain as a source of new natural products. Most of the compounds identified in the extract were previously isolated from marine *Streptomyces*. An extensive literature search on the biological activities of these compounds revealed that none of them have been tested/reported to have anti-biofilm effects, while several of the putative compounds identified are known to possess anti-fungal, anti-cancer, anti-mycobacterial and anti-malarial effects. This further highlights the novelty in discovery of compound(s) with specific anti-biofilm effects from the SBT343 extract. Up-scaling of the fermentation process and consequent bio-assay guided fractionation would help in isolation and identification of active compound(s) in the extract. In conclusion, our results show that the chemically rich *Streptomyces* sp. SBT343 extract has the potential to prevent the staphylococcal biofilm formation on polystyrene, glass, and contact lens surface without exhibiting toxic effects on bacterial and mammalian cells. Future characterization of lead compounds in this extract may yield novel anti-biofilm compound(s) of pharmaceutical interest.

## Author Contributions

Conceived and designed the experiments: UA, TÖ, UH, WZ. Performed the experiments: SB, EO. Analyzed the data: SB, EO, TÖ, UA. Manuscript preperation: SB, EO, DK, UA, HS, UH, WZ, TÖ. Manuscript revision: SB, EO, HS, UA, UH, WZ, TÖ. All authors read and approved the final manuscript.

## Conflict of Interest Statement

The authors declare that the research was conducted in the absence of any commercial or financial relationships that could be construed as a potential conflict of interest.

The reviewer LC and handling Editor declared their shared affiliation, and the handling Editor states that the process nevertheless met the standards of a fair and objective review.
