# Supplementary material for: Marine Sponge-Derived Streptomyces sp. SBT343 Extract Inhibits Staphylococcal Biofilm Formation
Source: Front Microbiol. 2017 Feb 16;8:236. doi: 10.3389/fmicb.2017.00236 (PMC5311426; doi:10.3389/fmicb.2017.00236)
Supplement: Supplementary file 1 [file Image_1.pdf]

Supplementary Material

Marine sponge-derived *Streptomyces* sp. SBT343 extract inhibits staphylococcal biofilm formation

Srikkanth Balasubramanian, Eman Maher Othman, Daniel Kampik , Helga Stopper, Ute Hentschel, Wilma Ziebuhr, Tobias A. Oelschlaeger* and Usama Ramadan Abdelmohsen*

**** Correspondence:*** *Corresponding Author:* usama.ramadan@uni-wuerzburg.de (UA)
t.oelschlaeger@uni-wuerzburg.de (TÖ)

Supplementary Figure

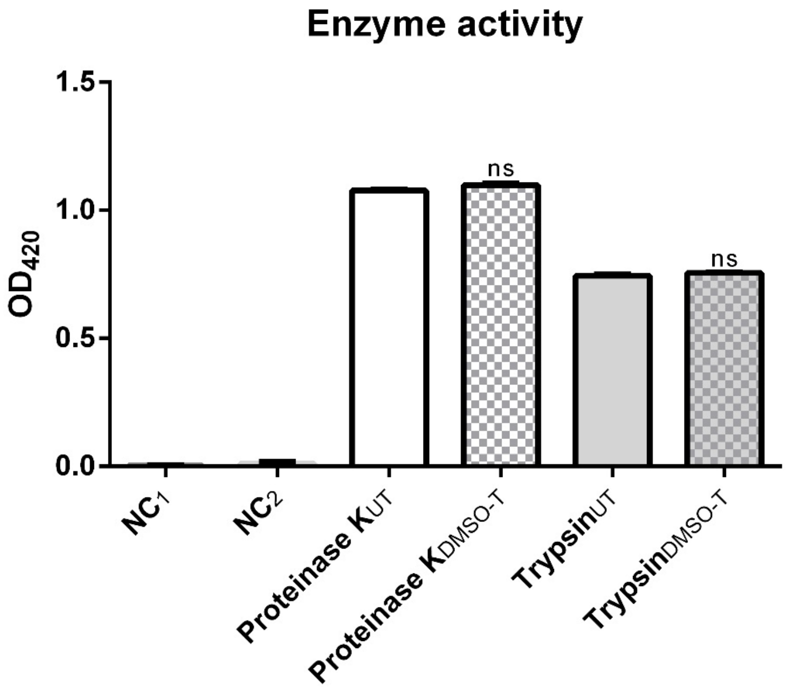

Supplementary Figure 1. Enzyme activity as determined by azocasein assay. OD_{420} values are directly proportional to the enzyme activities. NC1, negative control 1 (100 mM Tris pH 7.5); NC2, negative control 2 (100 mM Tris pH 7.5 + DMSO); UT, untreated; DMSO-T, DMSO treated. Each data point is composed of three independent experiments and standard errors are reported.
